# Highly Efficient and Eco-Friendly Thermal-Neutron-Shielding Materials Based on Recycled High-Density Polyethylene and Gadolinium Oxide Composites

**DOI:** 10.3390/polym16081139

**Published:** 2024-04-18

**Authors:** Donruedee Toyen, Ekachai Wimolmala, Kasinee Hemvichian, Pattra Lertsarawut, Kiadtisak Saenboonruang

**Affiliations:** 1 Department of Materials Science, Faculty of Science, Kasetsart University, Bangkok 10900, Thailand; donruedee.toyen@ku.th; 2 Special Research Unit of Radiation Technology for Advanced Materials (RTAM), Faculty of Science, Kasetsart University, Bangkok 10900, Thailand; 3 Polymer PROcessing and Flow (P-PROF) Research Group, Division of Materials Technology, School of Energy, Environment and Materials, King Mongkut’s University of Technology Thonburi, Bangkok 10140, Thailand; ekachai.wim@kmutt.ac.th; 4 Nuclear Technology Research and Development Center, Thailand Institute of Nuclear Technology (Public Organization), Nakhon Nayok 26120, Thailand; kasinee@tint.or.th (K.H.); pattra@tint.or.th (P.L.); 5 Department of Applied Radiation and Isotopes, Faculty of Science, Kasetsart University, Bangkok 10900, Thailand; 6 Specialized Center of Rubber and Polymer Materials in Agriculture and Industry (RPM), Faculty of Science, Kasetsart University, Bangkok 10900, Thailand

**Keywords:** recycled materials, neutrons, attenuation, rare-earth oxide, mechanical properties, gamma aging

## Abstract

Due to the increasing demands for improved radiation safety and the growing concerns regarding the excessive use of plastics, this work aimed to develop effective and eco-friendly thermal-neutron-shielding materials based on recycled high-density polyethylene (r-HDPE) composites containing varying surface-treated gadolinium oxide (Gd_2_O_3_) contents (0, 5, 10, 15, and 20 wt%). The results indicate that the overall thermal-neutron-shielding properties of the r-HDPE composites were enhanced with the addition of Gd_2_O_3_, as evidenced by large reductions in I/I_0_, HVL, and TVL, as well as the substantial increases in ∑_t_ and ∑_t/ρ_ of the composites. Furthermore, the results reveal that the values for tensile properties initially increased up to 5–15 wt% of Gd_2_O_3_ and then gradually decreased at higher contents. In addition, the results show that the addition of Gd_2_O_3_ particles generally increased the density (ρ), the remaining ash at 600 °C, and the degree of crystallinity (%XC) of the composites. This work also determined the effects of gamma irradiation on relevant properties of the composites. The findings indicate that following gamma aging, the tensile modulus slightly increased, while the tensile strength, elongation at break, and hardness (Shore D) showed no significant (*p* < 0.05) differences, except for the sample containing 5 wt% of Gd_2_O_3_, which exhibited a noticeable reduction in elongation at break. Furthermore, by comparing the neutron-shielding and mechanical properties of the developed r-HDPE composites with common borated polyethylene (PE) containing 5 wt% and 15 wt% of boron, the results clearly indicate the superior shielding and tensile properties in the r-HDPE composites, implying the great potential of r-HDPE composites to replace virgin plastics as effective and more eco-friendly shielding materials.

## 1. Introduction

Radiation technologies, particularly those involving neutrons, have seen major advancements and widespread application across various fields. These applications include neutron imaging in materials science and medicine [1,2], Boron Neutron Capture Therapy (BNCT) for treating brain cancers [3], the production of radioisotopes for medical diagnostics [4], power generation through nuclear fission and nuclear fusion [5,6], and moisture measurements in field applications [7]. These technologies not only enhance product quality, but often also present more environmentally friendly alternatives to conventional methods [8]. Nevertheless, it is crucial to acknowledge that excessive exposure to ionizing radiation can pose severe risks, especially to humans, potentially leading to organ abnormalities and even loss of life [9,10]. Consequently, strict safety protocols must be followed by all nuclear and radiation-related facilities to ensure the well-being of personnel, users, and the surrounding public [11].

Within the framework of the “ALARA” principle (As Low As Reasonably Achievable), one of the three fundamental approaches for bolstering the safety of relevant users involves the utilization of efficient and appropriate shielding equipment. The choice of materials and designs for such equipment depends on various factors, including the type and energy of the radiation, space constraints, and the intended application [12,13]. Particularly for neutron shielding equipment, commonly used materials include natural and synthetic rubbers (NR and SR, respectively), polyethylene (PE), paraffin, and epoxy [14,15,16]. These materials are suitable for such purposes due to their high contents of lightweight elements, particularly hydrogen (H) and carbon (C), within their chemical compositions. These elements efficiently scatter incoming neutrons, leading to substantial energy losses and necessitating fewer scatters to sufficiently lower neutron intensities compared to those containing heavier elements [17].

Although the previously mentioned polymers can effectively reduce neutron intensity through elastic scattering, their efficiency in shielding, particularly against thermal neutrons, can be substantially enhanced by incorporating them with compounds having a high neutron absorption cross section (σ_abs_), such as boron carbide (B_4_C), boron oxide (B_2_O_3_), boron nitride (BN), and boric acid (H_3_BO_3_) [18,19,20,21]. The addition of boron compounds introduces a more efficient and superior mechanism for attenuating thermal neutrons through neutron absorption, resulting in less materials being needed to achieve adequate safety and protection. Some examples of the utilization of boron compounds in neutron shielding include the addition of BN into ultrahigh molecular weight polyethylene fiber (UPEF) and polyurethane (PU) composites, where the results indicated a substantial increase in the linear attenuation coefficient (µ) values, rising from 6 cm^−1^ in UPEF/PU composites to 22 cm^−1^ in 20 wt% BN/UPEF/PU composites, producing a roughly 3.5-fold improvement [22]. Another example involves the addition of B_2_O_3_ into wood/NR composites, which resulted in enhanced attenuating capabilities of the materials, as shown by a reduction in neutron transmission ratios (I/I_0_) from 95.6% in a 2.5 mm thick wood/NR sample to 60.2% in a 2.5 mm thick sample containing 80 parts per hundred parts of rubber by weight (phr), producing a roughly nine-fold improvement [23,24].

Apart from boron compounds, there has been major interest among researchers in utilizing rare-earth oxides, particularly gadolinium oxide (Gd_2_O_3_), as potential substitutes for traditional boron compounds. This is due to gadolinium (Gd) offering a substantially higher σ_abs_ than that of boron (B), with the σ_abs_ values for Gd and B being 49,700 and 767 barns, respectively [25], making Gd_2_O_3_ a superior thermal neutron absorber than boron compounds at the same content [26]. In addition to its effectiveness as a neutron absorber, Gd also has a relatively high atomic number (Z) of 64, as well as a high density (ρ) of Gd_2_O_3_ (ρ = 7.41 g/cm^3^), making the compound well suited for attenuating gamma rays through photoelectric absorption, Compton scattering, and pair production [26,27]. Consequently, materials containing Gd_2_O_3_ can simultaneously attenuate both thermal neutrons and secondary gamma rays that are often produced following neutron absorption, distinguishing them from boron-based composites by eliminating the need for additional layers of lead (Pb) or other heavy-metal materials as gamma shielding [28,29].

In addition to the choice of fillers, main materials also play pivotal roles in defining the properties and practical utility of the products. While there is a range of materials available for manufacturing neutron-shielding products, HDPE ((C_2_H_4_)_n_) stands out as a prevalent and efficient polymer for applications that require strength and rigidity, such as construction parts, partitions, and containers for radioactive sources, due to its exceptional strength, durability, and cost-effectiveness [30], as well as its high carbon (C) and hydrogen (H) contents, which are suitable for neutron scattering [31]. Nevertheless, the growing demands for HDPE, both in households and industries, have raised serious concerns regarding the plastic pollution in oceans and landfills that severely harm humans, animals, plants, and the environment in general [32]. As a result, this drawback from the increasing utilization of HDPE has hindered its usefulness and underscored the need to reduce the accumulation of HDPE in the environment through recycling process so that the recycled products/tools can be sufficiently and safely used again [33,34]. Specifically for recycled HDPE (r-HDPE) in radiation protection, there have been several works aimed at creating effective shielding products that yielded promising outcomes and impacts. For example, r-HDPE was incorporated with lead oxide (PbO) nanoparticles for gamma protection, with the results demonstrating that the composites could effectively attenuate gamma rays at energies of 59.53, 661.66, 1173.25, and 1332.50 keV, as evidenced by the high values of the linear attenuation coefficient (µ) of 5.760, 0.241, 0.146, and 0.134 cm^−1^, respectively, as determined at 50 wt% PbO [35]. This example not only underscores the great potential of utilizing r-HDPE in radiation protection, but also offers a sustainable alternative to virgin plastics that would conserve valuable natural resources, as well as reduce energy consumption and greenhouse gas emissions during production [36].

To further advance the progress in the development of radiation-shielding products using recycled materials and to expand their potential applications to neutron shielding, the present research determined the optimum formulation and process for the preparation of Gd_2_O_3_ particles through a surface treatment using a silane coupling agent (3-aminopropyltriethoxysilane; KBE903), by considering the effects of KBE903 on the mechanical and neutron-shielding properties of the composites [37]. Furthermore, with the optimal silane content determined, varying contents of treated Gd_2_O_3_, (0, 5, 10, 15, and 20 wt%) were incorporated into r-HDPE, and important properties of interest were comprehensively examined and compared in terms of the following: thermal neutron shielding (the total macroscopic cross section (Σ_t_), the mass attenuation coefficient (∑_t,ρ_), the half-value layer (HVL), and the tenth value layer (TVL)), mechanical (tensile modulus, tensile strength, elongation at break, and hardness (Shore D)), morphological (SEM images), physical (density), and thermal (thermogravimetric analysis) properties. Additionally, since degradation due to exposure to radiation during actual use was possible, changes in the mechanical and thermal-neutron-shielding properties of the composites under 70 kGy gamma aging were determined and compared with those of non-aged samples. The outcomes of this research should not only provide in-depth insights into the advancement of enhanced thermal-neutron-shielding materials using r-HDPE composites with the addition of rare-earth oxides, but also elucidate the optimal processes for sample preparation involving surface treatment, which could potentially maintain or even enhance specific properties of the composites.

## 2. Materials and Methods

### 2.1. Surface Treatment of Gd_2_O_3_ Particles

**Figure 1:**
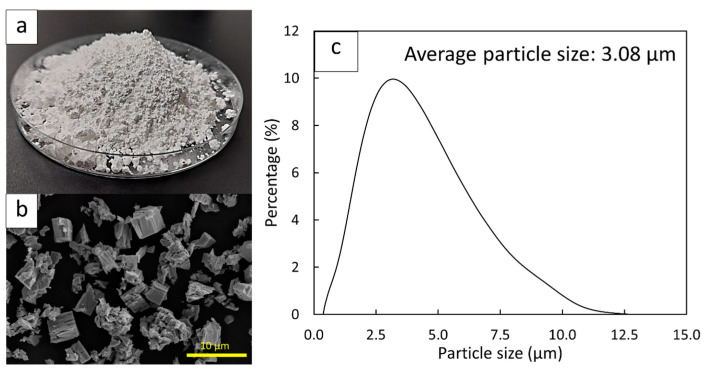
(**a**) Optical and (**b**) micrograph images of Gd_2_O_3_ particles and (**c**) distribution of Gd_2_O_3_ particle sizes.

The Gd_2_O_3_ particles were acquired from the Richest Group (Shanghai, China). Figure 1a,b show optical and micrograph images, respectively, of Gd_2_O_3_, illustrating the colors, shapes, and sizes of the particles used in this work. The distribution of particle sizes, determined using a laser diffraction technique (Mastersizer-2000; Malvern Instruments Limited; Malvern, UK), with a particle size analysis range of 0.02–15 µm, is shown in Figure 1c, revealing an average particle size of 3.08 µm.

Due to the poor interfacial compatibility between the HDPE and Gd_2_O_3_ surfaces [38], the Gd_2_O_3_ particles were surface-treated using a silane coupling agent (3-aminopropyltriethoxysilane; KBE903) prior to compounding with the r-HDPE samples. To initiate the treatment procedure, KBE903, ethanol, and distilled water, with their respective contents shown in Table 1, were thoroughly mixed for 15 min using a magnetic stirrer (RSM-01; Phoenix; Mannheim, Germany). Subsequently, the Gd_2_O_3_ particles were introduced into the mixture and stirred for an additional 30 min at a rotational speed of 1000 rpm. Then, the mixture was transferred to a water bath, and the stirring continued for 1 h at a water temperature of 90 °C. After stirring, the mixture was oven dried (ED/FD; Binder; Tuttlingen, Germany) at 80 °C for 3 h to eliminate any residual ethanol and distilled water, before being stored in sealed containers for subsequent processing and characterization.

### 2.2. Preparation of Gd_2_O_3_/r-HDPE Samples

**Figure 2:**
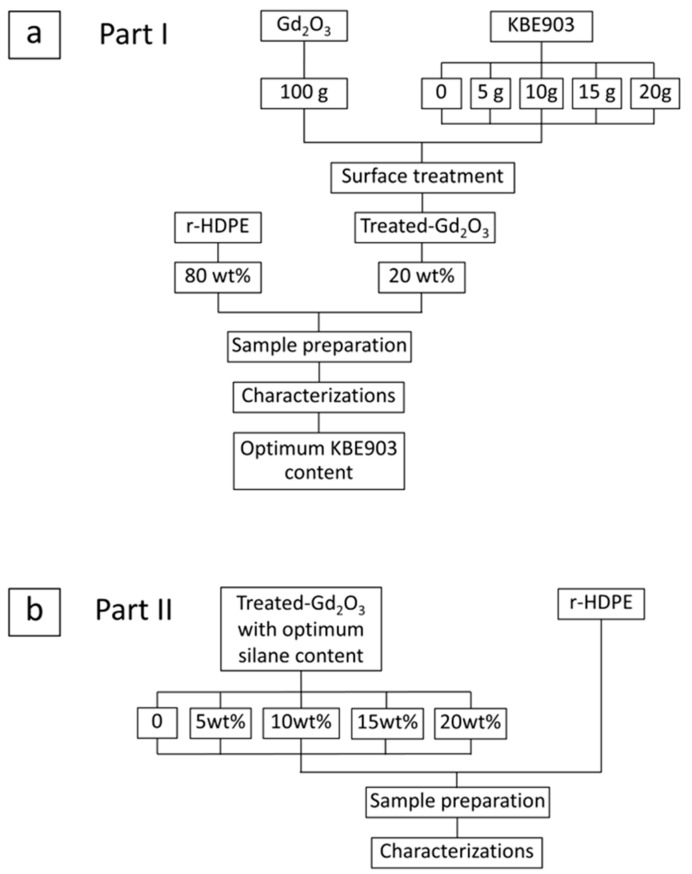
Scope of work showing (**a**) Part I, optimization procedure for surface treatment of Gd_2_O_3_ particles using silane coupling agent (KBE903), and (**b**) Part II, preparation and investigation of properties for Gd_2_O_3_/r-HDPE composites with varying Gd_2_O_3_ contents.

r-HDPE granules, with a melt flow index of 0.55 g/10 min, was kindly provided by S.P. Plastic Industry (Bangkok, Thailand). To prepare the samples, r-HDPE and treated Gd_2_O_3_, with contents as shown in Table 2, were continuously mixed using a high-speed mixer (LMXZ; Lab Tech Engineer; Samut Prakan, Thailand) and then compounded using a twin-screw extruder (CTW 1000C; Haake Rheomax; Leipzig, Germany), with the temperatures for feed, plastification, mixing, and die zones being 175, 180, 185, and 190 °C, respectively, at a screw speed of 60 rpm. The Gd_2_O_3_/r-HDPE samples with dimensions of 18 cm × 9 cm and a thickness of 2 mm were formed by molding the granules using a hot press (LP-20M LMXS; Lab Tech Engineering; Samut Prakan, Thailand) at a pressure of 15 MPa and a temperature of 190 °C for 8 min. Notably, the content of Gd_2_O_3_ was kept at 20 wt% (the maximum content of Gd_2_O_3_ investigated in this work) during the silane optimization procedure (Part I). Following the completion of Part I, the optimum KBE903 content was obtained and subsequently used for the preparation of samples with varying Gd_2_O_3_ contents from 0 to 20 wt% (Part II). Figure 2a,b illustrate the overall scope of this work, including Parts I and II, respectively.

### 2.3. Gamma Irradiation on Gd_2_O_3_/r-HDPE Samples

To understand the effects of gamma irradiation (gamma aging) on the properties of Gd_2_O_3_/r-HDPE composites, all samples were gamma irradiated with a total dose of 70 kGy using a gamma irradiator equipped with a ^60^Co source (Ob-servo ignis-09; Institute of Isotopes Co., Ltd.; Budapest, Hungary) with a dose rate of 9.2 kGy/h at the Thailand Institute of Nuclear Technology (Public Organization). Then, the changes in the thermal-neutron-shielding and mechanical properties for all samples under gamma aging were compared with those of non-aged samples.

### 2.4. Characterization

#### 2.4.1. Thermal-Neutron-Shielding Properties

A narrow-beam neutron source (47-Ci ^241^Am/Be) enclosed in a paraffin container was used. A stack of 5 cm thick HDPE sheets positioned in front of the r-HDPE samples served as a moderator to thermalize the 4.5-MeV neutrons from the source. Then, the transmitted neutrons were detected using a cylindrical ^3^He neutron detector (Model 251; LND; Oceanside, NY, USA) with a diameter of 12.7 mm. The detector was connected to a high-voltage power supply (Model 659; Ortec; Atlanta, GA, USA), a preamplifier/amplifier (Model 2022; Canberra; Atlanta, GA, USA), and a counter (Model 535P; Tennelec; Oak Ridge, TN, USA). For each formulation, 5 independent tests, each lasting 60 s, were conducted, and the average counts (I) of the transmitted neutrons were recorded. Additionally, the initial count (I_0_) was recorded, representing the neutron count in the absence of any samples. The determination of thermal-neutron-shielding parameters consisted of neutron transmission ratios (I/I_0_), the total macroscopic cross section (Σ_t_), the mass attenuation coefficient (∑_t/ρ_), the half-value layer (HVL), and the tenth value layer (TVL). The corresponding equations for the calculations of these parameters as well as the graphical setup of the neutron shielding tests are available elsewhere [17].

#### 2.4.2. Density and Degree of Crystallinity

The densities for all Gd_2_O_3_/r-HDPE composites were measured using a densitometer (MH-300A; Shanghai, China) with a precision of 0.01 g/cm^3^, following Archimedes’ principle [39] and ASTM D792-13 standard testing [40]. In addition, the theoretical densities (ρ_t_) of the Gd_2_O_3_/r-HDPE composites were determined to identify any discrepancies between the calculated and actual densities. The theoretical densities were calculated using Equations (1) and (2) [27]:

(1) ρt=100Cr-HDPEρr-HDPE+CGd2O3ρGd2O3

where C_r-HDPE_, C_Gd_2_O_3__, ρ_r-HDPE_, and ρ_Gd_2_O_3__ are the r-HDPE content (wt%), the Gd_2_O_3_ content (wt%), the density of r-HDPE (0.93 g/cm^3^), and the density of Gd_2_O_3_ (7.41 g/cm^3^), respectively.

In addition, the degree of crystallinity (%XC) of the composites, which represents the percentage of crystalline regions in the sample, was assessed using X-ray diffraction (XRD; D8 Advance; Bruker; Mannheim, Germany) with Ni-filtered CuKα radiation within a scanning angle range of 10°–80° (2θ) and a scanning speed of 0.02°/step. The %XC was determined by finding the ratio of the area under the crystalline peak (A_C_) to the sum of the area of all crystalline and amorphous peaks (A_T_) as shown in Equation (2) [41,42]:

(2) $$\%XD=\frac{A_{C}}{A_{T}}\times 100\%$$

#### 2.4.3. Morphology and Elemental Composition Analysis

The determination of morphology, the dispersion of Gd_2_O_3_ particles, the distribution of Gd elements in the r-HDPE matrix, and the elemental composition of the Gd_2_O_3_/r-HDPE composites was conducted using scanning electron microscopy (SEM) (Quanta 450; FEI; Brno-Černovice, Czech Republic) and energy-dispersive X-ray (EDX) spectroscopy (X-Max; Oxford Instruments; Abingdon, UK). For the determination of the morphologies of the fractured surfaces, all samples were immersed in liquid nitrogen for 5 min and then abruptly snapped. Notably, all specimens were coated with a 0.2 mm thick layer of gold using a magnetron sputter (SC7620; Quorum Technologies Polaron; Hertfordshire, UK) at a current of 5 mA for 120 s prior to the SEM-EDX investigations in order to improve electrical conductivity on the sample surface to avoid effects of charge accumulation that may distort or reduce image quality.

#### 2.4.4. Functional Groups and Thermal Properties

The identification of active functional groups presented on both the untreated and treated Gd_2_O_3_ particles was carried out using Fourier-transform infrared (FTIR) spectroscopy; (Vertex 70; Bruker; Billerica, MA, USA), covering wavenumbers within the range 400–4000 cm^−1^. The thermal properties of all the Gd_2_O_3_/r-HDPE composites were determined through thermalgravimetric analysis (TGA; TGA/DSC2/LF/1100; Mettler Toledo; Greifensee, Switzerland), with 0.4 mg of each sample enclosed in an aluminum oxide crucible and subjected to heating from room temperature to 800 °C, at a scanning rate of 20 °C/min (under a nitrogen atmosphere).

#### 2.4.5. Mechanical Properties

The mechanical properties, consisting of the tensile modulus, tensile strength, and elongation at break, for all the Gd_2_O_3_/r-HDPE composites were assessed using a Universal Testing Machine (TM-G5K; TM Tech Testing Co., Ltd.; Bangkok, Thailand). The testing procedure adhered to the ASTM D638-14 standard [43] testing at a testing speed of 50 mm/min. For the surface hardness measurements, the samples were tested using a durometer (GS-719G; Teclock; Nagano, Japan) according to ASTM D2240–03 [44] (Shore D) standard testing. Notably, a minimum of 3 repetitions for each formulation was carried out for all mechanical measurements.

### 2.5. Statistical Analysis

A level of 95% significance (*p* < 0.05) was used for the descriptive analysis of the data. A Student’s *t*-test was also applied to determine any significant differences between the results of interest. The statistical analyses were conducted using the IBM SPSS Statistics 20 software (New York, NY, USA) [17].

## 3. Results and Discussion

### 3.1. Optimization for Surface Treatment of Gd_2_O_3_ Particles

#### 3.1.1. Functional Groups

**Figure 3:**
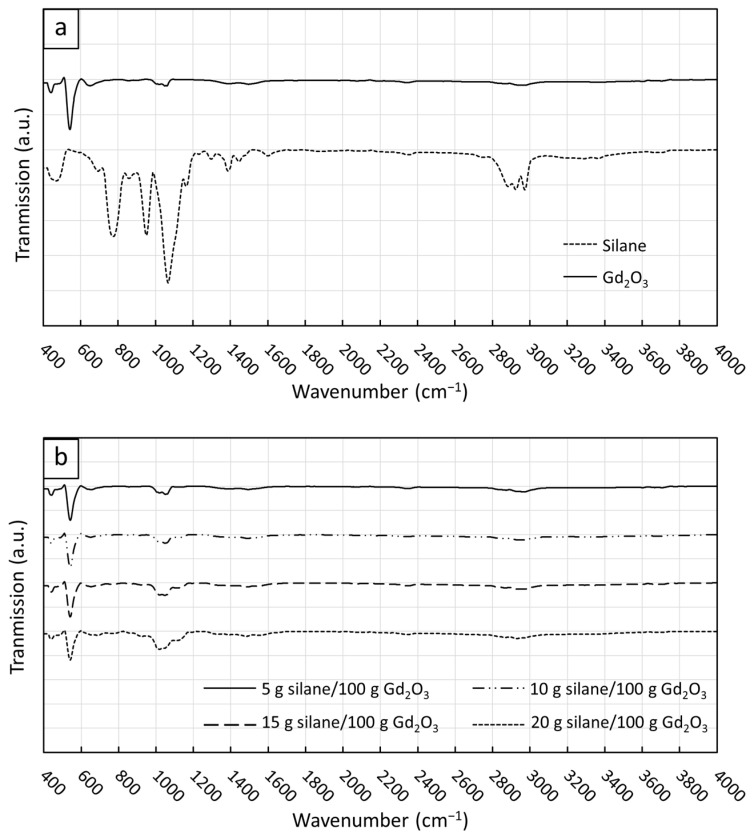
Functional groups determined using FTIR of (**a**) silane coupling agent (KBE903) and non-treated Gd_2_O_3_ particles and (**b**) treated Gd_2_O_3_ particles with varying KBE903 contents (5, 10, 15, and 20 g/100 g Gd_2_O_3_).

The functional groups of KBE903, non-treated Gd_2_O_3_, and treated Gd_2_O_3_ particles, determined using FTIR, are shown in Figure 3. For the spectra of KBE903 (Figure 3a), dominant peaks were observed at 475 cm^−1^ (Si–O–Si), 700 cm^−1^ (C–H), 770 cm^−1^ (Si–O–C), 865 cm^−1^ (Si–O), 950 cm^−1^ (Si–O), 1066 cm^−1^ (Si–O), 1167 cm^−1^ (Si–O–C), 1300 cm^−1^ (C–O), 1390 cm^−1^ (O–H), 1450 cm^−1^ (C–H), 1600 cm^−1^ (N–H), and 2880–2980 cm^−1^ (C–H) [45,46], while dominant peaks for the non-treated Gd_2_O_3_ were observed at 445 cm^−1^ and 545 cm^−1^, corresponding to the stretching vibration of Gd_2_O_3_, and 1020–1060 cm^−1^, corresponding to the stretching vibrations of Gd–O bonds [37]. For the treated Gd_2_O_3_ particles, the FTIR spectra (Figure 3b) showed an additional broad peak at 1116 cm^−1^ (Si–O–C) (in addition to peaks at 445 cm^−1^, 545 cm^−1^, and 1020–1060 cm^−1^) with their amplitudes increasing with the KBE903 contents. These additional peaks, as well as the observed correlation between peak amplitudes and KBE903 contents, clearly imply the successful treatment of Gd_2_O_3_ particles by grafting KBE903 on the surfaces of Gd_2_O_3_ [47]. It should be noted that there was a shift in the Si–O–C spectrum from 1167 cm^−1^ in KBE903 to 1116 cm^−1^ in the treated Gd_2_O_3_ particles, which could possibly be due to the effects of hydrolysis during the treatment process that changed the degree of hydrolysis and condensation of KBE903, influencing the Si–O–C peak positions in the composites [48]. Another plausible explanation of the shift in the FTIR spectra could be due to the merger between the 1066 cm^−1^ (Si–C) and 1167 cm^−1^ (Si–O–C) peaks of KBE903, resulting in a new broader peak at 1116 cm^−1^.

#### 3.1.2. Density and Elemental Composition

The densities of the 20 wt% Gd_2_O_3_/r-HDPE composites are shown in Table 3; there were no significant differences among the densities for all samples, with the values being in the range 1.11–1.13 g/cm^3^. These measured densities were in good agreement with the theoretical value of 1.13 g/cm^3^, derived using Equation (1), with slight differences possibly due to the presence of voids in the r-HDPE matrix that reduced the final mass of the samples.

Table 3 also shows the elemental composition of the 20 wt% Gd_2_O_3_/r-HDPE composites, with varying KBE903 contents (0–20 g/100 g Gd_2_O_3_). The results indicate that the compositions of Gd for all samples were not significantly different, with the values being in the range 19.98–22.33% (by weight). On the other hand, the compositions of Si increased with increasing KBE903 contents, implying the presence of KBE903 in the composites and a successful surface treatment of Gd_2_O_3_ particles.

#### 3.1.3. Thermal-Neutron-Shielding Properties

**Figure 4:**
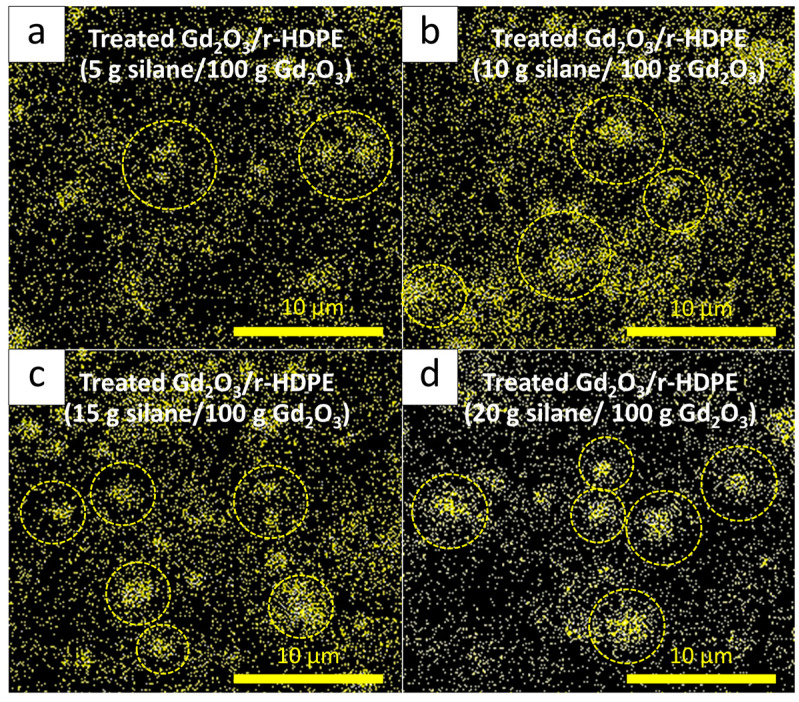
SEM-EDX mapping showing the distribution of Gd elements in the 20 wt% Gd_2_O_3_/r-HDPE composites, with varying silane (KBE903) contents of (**a**) 5 g, (**b**) 10 g, (**c**) 15 g, and (**d**) 20 g per 100 g of Gd_2_O_3_. Dotted circles indicate areas with Gd agglomeration.

The thermal-neutron-shielding properties, consisting of I/I_0_, ∑_t_, ∑_t/ρ_, and HVL, for all 20 wt% Gd_2_O_3_/r-HDPE samples are shown in Table 4, which indicate that the sample containing Gd_2_O_3_ treated with 5 g of KBE903 per 100 g of Gd_2_O_3_ had the highest thermal-neutron-shielding properties compared to those treated with other KBE903 contents, as evidenced by the lowest values for I/I_0_ and HVL, and the highest values of ∑_t_ and ∑_t/ρ_ in the former. These differences in the shielding abilities for each condition could be explained by the distribution of Gd elements in the composites, as illustrated in Gd mapping images (Figure 4), which clearly reveal substantially more uniform dispersion and distribution of Gd elements in the sample with 5 g of KBE903 (Figure 4a). Consequently, incoming thermal neutrons could better interact and be absorbed by the Gd elements in the former, subsequently leading to higher overall shielding properties for the materials [37]. Notably, more particle agglomerations were observed in Figure 4b–d due to the excessive use of KBE903 that led to the initiation of self-agglomeration or the poor distribution of Gd_2_O_3_ particles, contributing to the increases in the values of I/I_0_ for the samples with 10–20 g of KBE903 per 100 g of Gd_2_O_3_ [49]. The self-agglomeration observed in the case of samples with excessive silane could have been because the surface of Gd_2_O_3_ becoming saturated with silane molecules, leading to a multilayer formation of silane that acted as a bridge between Gd_2_O_3_ particles, subsequently facilitating the agglomerations of the particles [50].

#### 3.1.4. Mechanical Properties

**Figure 5:**
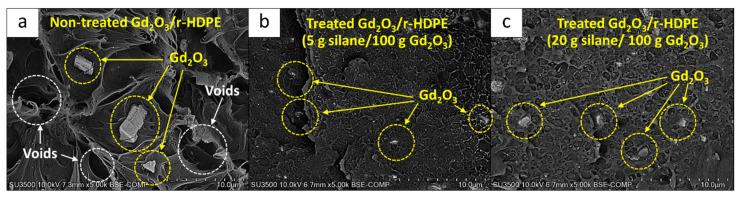
SEM images showing fractured surfaces of 20 wt% Gd_2_O_3_/r-HDPE composites with varying KBE903 contents of (**a**) 0, (**b**) 5 g, and (**c**) 20 g per 100 g of Gd_2_O_3_.

Table 5 presents the mechanical properties, consisting of the tensile modulus, tensile strength, elongation at break, and hardness (shore D), for the 20 wt% Gd_2_O_3_/r-HDPE composites, with the results showing a pattern similar to the thermal-neutron-shielding properties discussed in Section 3.1.3. Specifically, it was observed that the composites containing Gd_2_O_3_ treated with KBE903 with a content of 5 g/100 g Gd_2_O_3_ had the highest overall mechanical properties, as evidenced by their superior tensile modulus, tensile strength, and elongation at break compared to those under other conditions. This improvement in the mechanical properties of the former could have been due to the improved interfacial compatibility between the r-HDPE matrix and the treated-Gd_2_O_3_ particles, which resulted in the enhanced abilities of the materials to transfer and withstand external forces [51]. Figure 5, which shows the morphologies of the fractured surfaces of the samples, reveals visible voids, as well as clear Gd_2_O_3_ particles at the fractures, especially those without KBE903 treatment (Figure 5a) or with excessive KBE903 contents (Figure 5c). These observations indicate that the fractures occurred at voids or interfaces between the r-HDPE matrix and Gd_2_O_3_ particles, implying poor interfacial compatibility between the two substances [52]. On the other hand, Figure 5b shows that the Gd_2_O_3_ particles that were treated with 5 g of KBE903 per 100 g of Gd_2_O_3_, were mostly embedded/covered in the r-HDPE matrix at the fractured surface, implying better interfacial compatibility and hence higher overall values for the mechanical properties of the composites [53]. Notably, the hardness (Shore D) values of all samples were not significantly different, with the values being in the range 53–55.

As discussed in Section 3.1.3 and Section 3.1.4, it was evident that using a KBE903 content of 5 g/100 g Gd_2_O_3_ for the surface treatment of Gd_2_O_3_ resulted in the highest overall thermal-neutron-shielding and mechanical properties for the Gd_2_O_3_/r-HDPE composites (Table 4 and Table 5). Consequently, this particular KBE903 content, along with the developed surface treatment procedure, was chosen as the optimum condition for preparing Gd_2_O_3_ particles that were subsequently used for the preparation of Gd_2_O_3_/r-HDPE samples (with varying Gd_2_O_3_ contents) in Part II.

### 3.2. Effects of Gd_2_O_3_ Contents on Properties of Gd_2_O_3_/r-HDPE Composites

#### 3.2.1. Density and Elemental Composition of Gd_2_O_3_/r-HDPE Composites

The theoretical and measured densities of all Gd_2_O_3_/r-HDPE composites, with varying Gd_2_O_3_ contents (0, 5, 10, 15, and 20 wt%), are shown in Table 6. The results indicate that the densities of the samples increased with increasing Gd_2_O_3_ contents. This could have been due to the higher density of Gd_2_O_3_ than that of the r-HDPE, which resulted in an increased mass of the sample per unit volume after the addition of Gd_2_O_3_ (ρ_r-HDPE_ = 0.93 g/cm^3^ and ρ_Gd2O3_ = 7.41 g/cm^3^). Furthermore, it was found that the measured densities agreed with the theoretical ones, implying that the samples were well prepared. In addition, Table 6 shows the elemental compositions of the Gd_2_O_3_/r-HDPE composites, indicating that the composites were mainly composed of C, O, Si, and Gd (H was not detectable due to the detection limitation of SEM-EDX) [54]. Notably, the compositions of Gd were largely related to the nominal Gd_2_O_3_ contents, which partly confirmed that the samples were correctly prepared.

#### 3.2.2. Thermal Properties of Gd_2_O_3_/r-HDPE Composites

**Figure 6:**
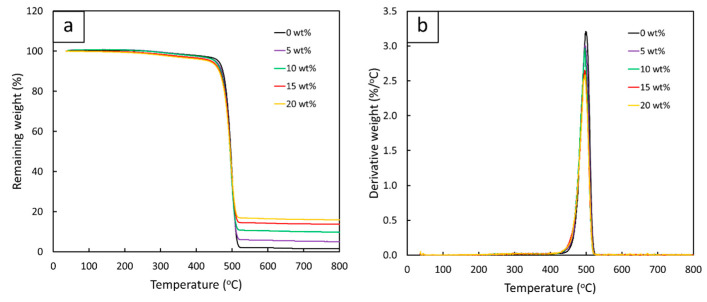
Thermal stability of Gd_2_O_3_/r-HDPE composites with varying Gd_2_O_3_ contents (0, 5, 10, 15, and 20 wt%), determined through TGA, with (**a**) correlations between remaining weight of r-HDPE composites and temperature and (**b**) correlations between derivative weight of r-HDPE composites and temperature.

The thermal properties of Gd_2_O_3_/r-HDPE composites with varying Gd_2_O_3_ contents (0, 5, 10, 15, and 20 wt%), determined through TGA, are shown in Figure 6. The results reveal that all samples had similar trends in weight loss and thermal stability, as illustrated in Figure 6a, suggesting that the addition of Gd_2_O_3_ had little impact on the thermal stability of the r-HDPE composites. In addition, the peaks of the derivative weights (Figure 6b) were in the range 493–495 °C, which corresponds to the decomposition of the r-HDPE matrix [55].

**Figure 7:**
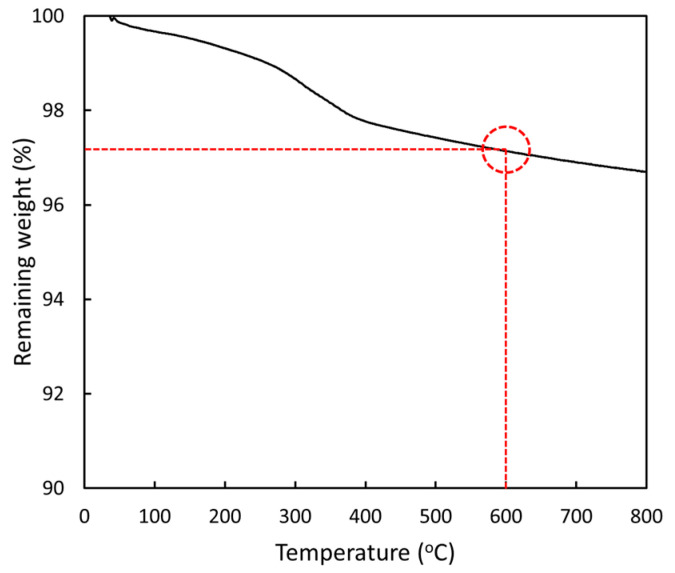
Thermal stability of Gd_2_O_3_, determined through TGA. The dotted circle indicates the remaining weight (%) of Gd_2_O_3_ particles at 600 °C.

The percentages of the remaining ash for all samples at 600 °C, derived from Figure 6a, are shown in Table 7, indicating that the amounts of remaining ash were fairly consistent with the nominal contents of the Gd_2_O_3_ added to the r-HDPE composites. This behavior was observed mainly due to the Gd_2_O_3_ having much higher decomposition temperatures (>600 °C), resulting in the majority of the Gd_2_O_3_ particles remaining at 600 °C. The high decomposition temperature of Gd_2_O_3_ was confirmed by the TGA results (Figure 7), with less than a 3% loss in the mass of Gd_2_O_3_ at 600 °C.

#### 3.2.3. Crystallinity of Gd_2_O_3_/r-HDPE Composites

The degrees of crystallinity (%XC) for all Gd_2_O_3_/r-HDPE samples, calculated from the plots of XRD (Figure S1), are shown in Table 8, indicating that the mean (±standard deviation) of %XC abruptly increased with the initial addition of 5 wt% Gd_2_O_3_ into the composites (increase from 38.6 ± 4.5% in the pristine r-HDPE to 53.6 ± 2.9% in the 5 wt% Gd_2_O_3_/r-HDPE composites) and then slightly decreased at higher contents. The initial increase in %XC could have been due to the high crystallinity of Gd_2_O_3_ particles that greatly increased the crystalline phase and subsequently increased the overall %XC of the composites. However, as more Gd_2_O_3_ particles were added to the samples, the mobility of the r-HDPE molecular chains was restricted due to the interference of Gd_2_O_3_ particles that retarded the growth in the crystalline phase (hindering the ability of the r-HDPE molecular chains to arrange themselves in an ordered crystalline structure) and subsequently lowered the %XC of the composites [17,56]. It should be noted that the crystalline peaks used for %XC calculations were 2θ = 10.3°, 22.4°, 24.8°, 27.6°, 29.4°, 33.9°, 48.2°, and 57.3°.

#### 3.2.4. Thermal-Neutron-Shielding Properties of Gd_2_O_3_/r-HDPE Composites

**Figure 8:**
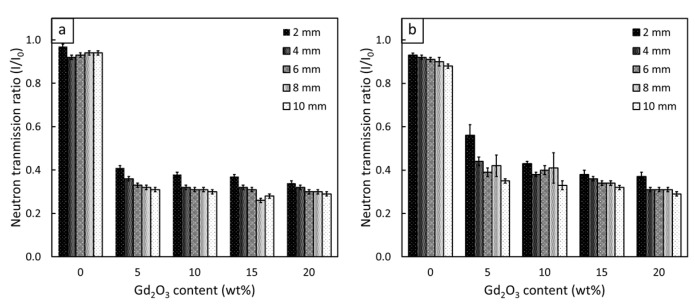
Neutron transmission ratios of (**a**) non-aged Gd_2_O_3_/r-HDPE composites and (**b**) gamma-aged Gd_2_O_3_/r-HDPE composites, with varying Gd_2_O_3_ contents and sample thicknesses, where error bars indicate ± standard deviation.

**Figure 9:**
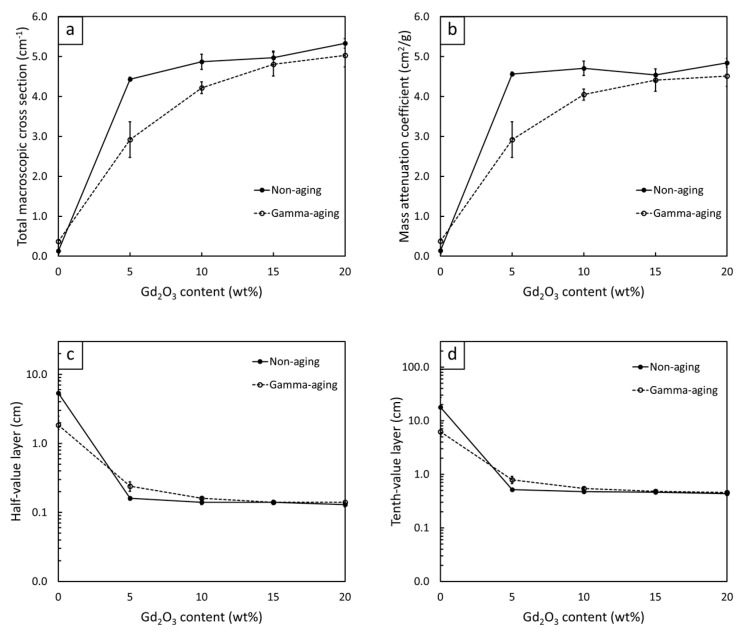
Thermal-neutron-shielding properties, consisting of (**a**) total macroscopic cross section (∑_t_), (**b**) mass attenuation coefficient (∑_t/ρ_), (**c**) half-value layer (HVL), and (**d**) tenth value layer (TVL) of non-aged Gd_2_O_3_/r-HDPE composites (solid lines) and gamma-aged Gd_2_O_3_/r-HDPE composites (dotted lines), where error bars indicate ± standard deviation.

**Figure 10:**
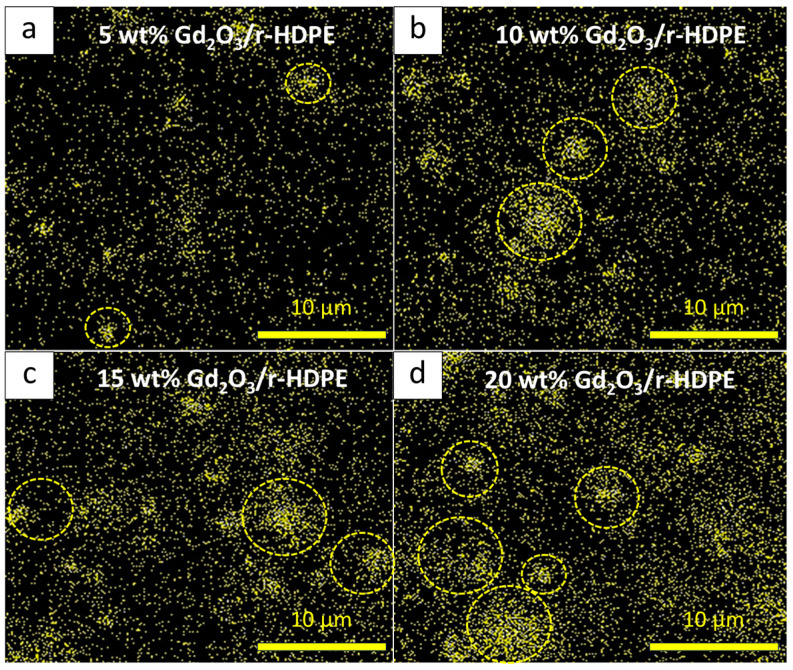
SEM-EDX mapping of Gd distribution in Gd_2_O_3_/r-HDPE composites with varying Gd_2_O_3_ contents of (**a**) 5 wt%, (**b**) 10 wt%, (**c**), 15 wt%, and (**d**) 20 wt%. Dotted circles indicate areas with visible Gd agglomeration.

The thermal-neutron-shielding properties of non-aged Gd_2_O_3_/r-HDPE composites with varying Gd_2_O_3_ contents and material thicknesses are shown in Figure 8a and Figure 9. The results indicate that the values of I/I_0_, HVL, and TVL were greatly reduced, while the values of ∑_t_ and ∑_t/ρ_ increased, when 5 wt% of Gd_2_O_3_ was initially introduced to the composites. For example, the values of I/I_0_ and HVL decreased from 0.98 and 5.38 cm, respectively, in the 2 mm r-HDPE sample to 0.41 and 0.16 cm, respectively, in the 2 mm r-HDPE sample containing 5 wt% Gd_2_O_3_, corresponding to an approximately 34-fold reduction in the materials needed to attenuate 50% of the initial neutron intensity. This pronounced improvement in the ability of the composites to attenuate thermal neutrons was primarily attributed to a shift in dominant neutron interactions from neutron scattering in the pristine r-HDPE sample to a more effective mechanism of neutron absorption in the Gd_2_O_3_/r-HDPE composites, due to the presence of Gd elements with a high value of σ_abs_ [57], consequently leading to the enhanced probability of thermal neutrons being absorbed by the Gd_2_O_3_/r-HDPE composites. Figure 8a and Figure 9 also show that by adding more Gd_2_O_3_ particles to the composites, the improvement in the shielding abilities of the composites became less pronounced, especially at higher (>15 wt%) Gd_2_O_3_ contents. This observed result could have been due to the existence of the neutron absorption mechanism at 5 wt% content, resulting in just a slight additional probability for thermal neutrons to be absorbed by the added Gd_2_O_3_ particles. Another possible explanation for this small improvement could be that the particle agglomeration and non-uniformity of the Gd distribution in the matrix (Figure 10), especially in the samples with high Gd_2_O_3_ contents (Figure 10c,d), limited the capability of the Gd elements to interact with neutrons [58]. Furthermore, Figure 8a reveals negative correlations between I/I_0_ and material thickness (x); hence, the ability of the samples to attenuate neutrons increased with increasing material thickness. This was observed primarily because there were a greater number of Gd elements per unit area of the sample that could interact and attenuate neutrons. In fact, this correlation was in agreement with the Beer–Lambert equation (Equation (3)) [59], which also depicts a negative relationship between I/I_0_ and x [37]:

(3) II0=e−∑t·x

Figure 8b and Figure 9 illustrate the thermal-neutron-shielding properties of the Gd_2_O_3_/r-HDPE composites after being gamma irradiated with a total dose of 70 kGy. The results indicate that there were slight decreases in the overall shielding properties of the gamma-aged samples, which might have been due to the initiation of chain scission and oxidative degradation caused by the gamma rays that altered the molecular structures and physical properties of the composites [60], resulting in more neutrons being able to transmit through the materials, leading to higher values for I/I_0_, HVL, and TVL (lower values for ∑_t_ and ∑_t/ρ_).

#### 3.2.5. Mechanical Properties of Gd_2_O_3_/r-HDPE Composites

**Figure 11:**
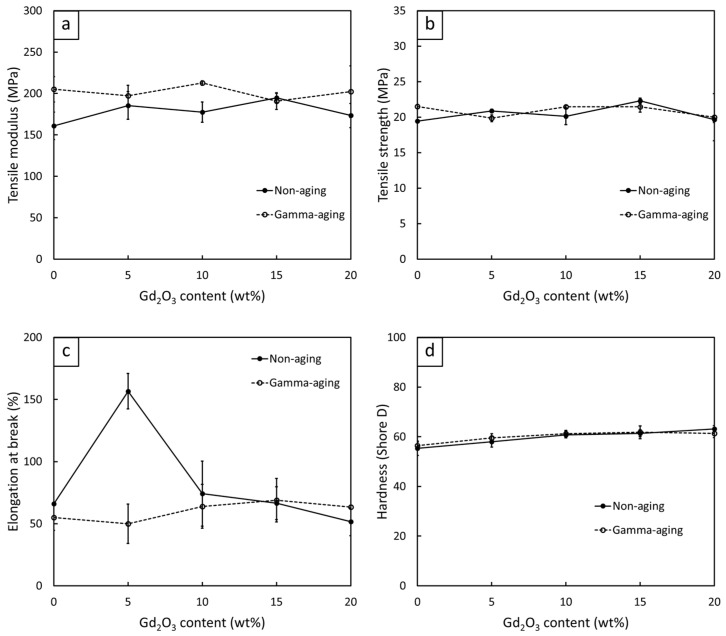
Mechanical properties, consisting of (**a**) tensile modulus, (**b**) tensile strength, (**c**) elongation at break, and (**d**) hardness (Shore D) of non-aged Gd_2_O_3_/r-HDPE composites (solid lines) and gamma-aged Gd_2_O_3_/r-HDPE composites (dotted lines), where error bars indicate ± standard deviation.

**Figure 12:**
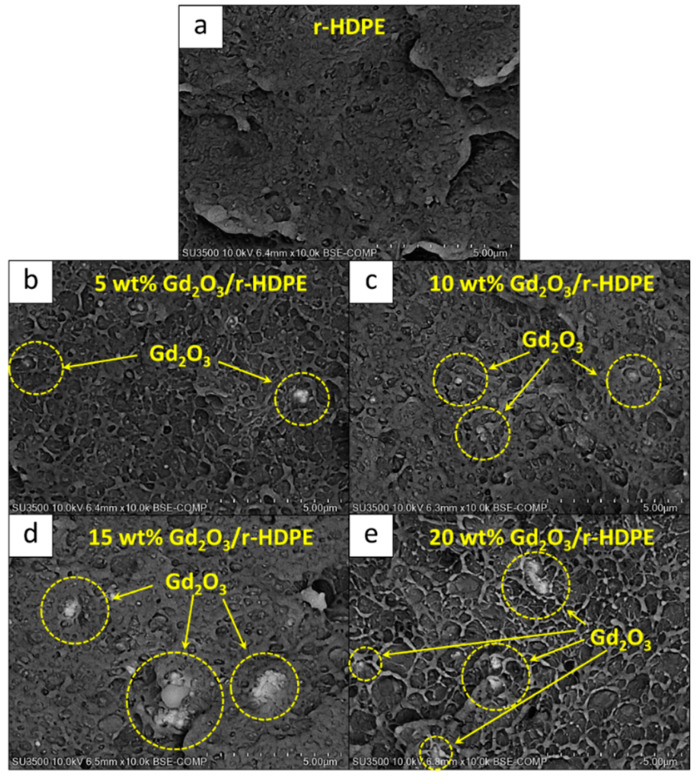
SEM images showing morphologies and particle distributions in (**a**) a pristine r-HDPE, and Gd_2_O_3_/r-HDPE composites with varying Gd_2_O_3_ contents of (**b**) 5 wt%, (**c**) 10 wt%, (**d**), 15 wt%, and (**e**) 20 wt%. Dotted circles indicate areas with visible particle agglomeration.

Figure 11 shows the mechanical properties, consisting of tensile modulus, tensile strength, elongation at break, and hardness (Shore D), of the non-aged and gamma-aged Gd_2_O_3_/r-HDPE composites with varying Gd_2_O_3_ contents (0, 5, 10, 15, and 20 wt%). The results indicate that the hardness (Shore D) gradually increased after the addition of Gd_2_O_3_, with the values rising up to 63 ± 1 for the sample containing 20 wt% Gd_2_O_3_ (compared to 55 ± 3 for the neat r-HDPE sample). The increase in hardness (Shore D) could have been due to the high rigidity of the Gd_2_O_3_ particles that subsequently increased the overall rigidity and hence the surface hardness of the composites [23]. Notably, the behaviors of tensile modulus and surface hardness for the samples with varying Gd_2_O_3_ contents were similar to those of the crystallinity shown in Table 8, which could be due to the crystalline regions within the sample tending to be more ordered and densely packed than those of amorphous regions, resulting in the samples with higher %XD exhibiting greater resistance to deformation and, hence, higher overall rigidity. On the other hand, the values of tensile modulus, tensile strength, and elongation at break behaved differently, with the values of tensile modulus/tensile strength increasing up to 15 wt% of Gd_2_O_3_ and elongation at break increasing up to 5 wt% of Gd_2_O_3_, and then gradually decreasing at higher Gd_2_O_3_ contents. These initial increases in the mentioned tensile properties could have been due to the improved filler dispersion within the r-HDPE matrix from the additional surface treatment, which subsequently improved the filler–matrix bonding at their interfaces and, subsequently, the abilities to transfer external forces from the matrix to the fillers [17]. However, as more Gd_2_O_3_ particles were added to the composites, there was noticeably higher particle agglomeration due to filler–filler interactions [61], as well as visible voids from the interfacial incompatibility between the Gd_2_O_3_ particles and the r-HDPE matrix (despite Gd_2_O_3_ being treated with KBE903) [62], resulting in the limited mobility of the r-HDPE molecular chains that restricted the deformation of the composites [17]. Micrographs illustrating the morphologies and particle distribution of all samples are shown in Figure 12, which reveals visible voids and agglomerations in the materials, especially in those with high filler contents (Figure 12d,e).

In addition, Figure 11 shows the changes in the mechanical properties of the Gd_2_O_3_/r-HDPE composites after being gamma irradiated (gamma aging). The results indicate that the tensile modulus values mostly increased after aging, which could have been due to the initiation of additional crosslinking between the r-HDPE molecular chains by gamma rays, which increased the overall rigidity and hence the tensile modulus of the composites [63]. Nonetheless, the values for tensile strength, elongation at break, and hardness (Shore D) for the non-aged and gamma-aged samples were not significantly different, except for the sample containing 5 wt% Gd_2_O_3_, which had a noticeable reduction in elongation at break after aging. The small changes in the tensile properties could have been due to the high atomic number of Gd, as well as the relatively high density of Gd_2_O_3_, which made Gd_2_O_3_ interact well with incoming gamma rays and subsequently suppress the effects of chain scission and oxidative degradation that would normally reduce the mechanical properties of the materials [27,64,65]. However, as previously mentioned, the elongation at break of the gamma-aged Gd_2_O_3_/r-HDPE composites with 5 wt% of Gd_2_O_3_ was significantly lower than that of the non-aged one. This could have been due to insufficient Gd_2_O_3_ content to suppress the degradation (chain scission) caused by gamma rays, resulting in reduced abilities of the composites to elongate along the direction of external forces.

### 3.3. Benchmarking Developed r-HDPE Composites with Commercial Borated Polyethylene (PE) Products

To determine the useability and to benchmark the developed r-HDPE composites, their thermal-neutron-shielding and mechanical properties were compared with those from commonly used neutron-shielding products based on borated PE composites that contain 5 wt% and 15 wt% of boron (B), as shown in Table 9. The results indicate that the overall thermal-neutron-shielding properties of the 5 wt% Gd_2_O_3_/r-HDPE composites had much higher values than those of the borated PE products (for both boron contents), as evidenced by the higher values of ∑_t_ and ∑_t/ρ_ and the lower values of HVL and TVL in the former. These substantially improved shielding properties could have been due to the much higher σ_abs_ value of Gd (49,700 barns) than that of B (767 barns) [25], resulting in a higher interaction probability between neutrons and Gd_2_O_3_ particles. Table 9 also shows that the commercial products were noticeably more brittle than the current r-HDPE composites, as seen in the lower tensile strength (as low as 16.6 MPa) and elongation at break (as low as 4%) in the former. The brittleness observed in the commercial products could have been due to the lower densities of boron compounds, such as boron carbide (2.52 g/cm^3^) or boron oxide (2.46 g/cm^3^), which led to an increased volume of fillers being added to the matrix to obtain the required boron contents, resulting in potentially more defects or particle agglomerations, or both, being observed in the matrix, which subsequently lowered the values of the tensile strength and elongation at break of the products.

As mentioned above, based on the overall comparisons between the developed 5 wt% Gd_2_O_3_/r-HDPE composites in this work and commercial PE products containing 5 wt% and 15 wt% of boron (B), it was evident that the r-HDPE composites had promising potential as effective thermal-neutron-shielding materials by offering highly efficient neutron attenuation as well as enhanced mechanical properties that widened the possible usage and application of the composites.

## 4. Conclusions

This research addressed the increasing demand for enhanced radiation safety and environmental concerns whilst addressing concerns about increasing plastic pollution by developing efficient and eco-friendly thermal-neutron-shielding materials using r-HDPE composites with varying contents of silane-treated Gd_2_O_3_. The results demonstrate that the addition of Gd_2_O_3_ significantly improved the thermal-neutron-shielding capabilities of the r-HDPE composites, as seen in the lower values for I/I_0_, HVL, and TVL and higher values of ∑_t_ and ∑_t/ρ_ after the addition of Gd_2_O_3_. Additionally, the mechanical properties, consisting of tensile modulus, tensile strength, and elongation at break, were initially enhanced with the addition of up to 5–15 wt% of Gd_2_O_3_, and then gradually declined at higher contents. Furthermore, the addition of Gd_2_O_3_ increased the values for surface hardness (Shore D), ρ, remaining ash at 600 °C, and the %XC of the composites. This study also investigated the impact of gamma irradiation on these composites, revealing a slight increase in tensile modulus, while tensile strength, elongation at break, and hardness (Shore D) remained relatively unaffected, except for in the 5 wt% Gd_2_O_3_ sample, which experienced a significant reduction in elongation at break. Furthermore, in comparing these r-HDPE composites with conventional neutron-shielding products based on PE composites containing 5 wt% and 15 wt% boron, our findings indicate that the r-HDPE composites had superior shielding and tensile properties than the latter, highlighting the potential of r-HDPE composites to replace virgin plastics as effective and more environmentally friendly shielding materials. Consequently, based on the overall results obtained, the developed r-HDPE composites could be used as alternative materials that not only offer highly effective thermal-neutron-shielding capability, but also provide a basis for the future development of high-valued recycled products.

## Figures and Tables

**Table 1:** Formulations, with names, contents, and suppliers for surface treatment of Gd_2_O_3_ particles.

Chemical	Content (g)	Supplier
Gd_2_O_3_	100	Richest Group (Shanghai, China)
Silane coupling agent (KBE903)	A *	Kisco(T) Ltd. (Bangkok, Thailand)
99% Ethanol	92 − A	Gammaco Co., Ltd. (Nonthaburi, Thailand)
Distilled water	8	Kasetsart University (Bangkok, Thailand)

* A = 0, 5, 10, 15, or 20.

**Table 2:** Formulations, with names, contents, and suppliers for preparation of Gd_2_O_3_/r-HDPE samples.

Chemical	Content (wt%)	Supplier
Gd_2_O_3_	B *	Richest Group (Shanghai, China)
r-HDPE	100 − B	S.P. Plastic Industry (Bangkok, Thailand)

* B = 0, 5, 10, 15, or 20.

**Table 3:** Densities and elemental compositions of 20 wt% Gd_2_O_3_/r-HDPE composites, with varying silane (KBE903) contents (0, 5, 10, 15, and 20 g/100 g Gd_2_O_3_). The values are shown as the mean ± standard deviation of the mean.

Silane Content (g/100 g Gd_2_O_3_)	Density (g/cm^3^)	Elemental Composition (% by Weight)			
		C	O	Si	Gd
0	1.11 ± 0.01	73.47 ± 4.32	6.11 ± 1.37	0.02 ± 0.01 *	20.41 ± 1.56
5	1.11 ± 0.02	74.37 ± 2.02	4.66 ± 1.29	0.06 ± 0.02	20.91 ± 0.73
10	1.13 ± 0.01	74.19 ± 2.74	4.30 ± 0.95	0.11 ± 0.06	21.40 ± 1.90
15	1.13 ± 0.02	73.47 ± 1.18	3.98 ± 1.13	0.23 ± 0.13	22.33 ± 1.88
20	1.12 ± 0.01	74.51 ± 2.60	5.25 ± 0.27	0.26 ± 0.05	19.98 ± 2.79

* The small content of Si in non-treated Gd_2_O_3_ could be due to a small contamination of silane in the equipment used during the treatment process. However, the Si content was small in that it had negligible effects for the determination of optimum silane content.

**Table 4:** Thermal-neutron-shielding properties, consisting of neutron transmission ratio (I/I_0_), total macroscopic cross section (∑_t_), mass attenuation coefficient (∑_t/ρ_), and half-value layer (HVL), of 20 wt% Gd_2_O_3_/r-HDPE composites, with varying silane (KBE903) contents (0, 5, 10, 15, and 20 g/100 g Gd_2_O_3_). The values are shown as the mean ± standard deviation of the mean.

Silane Content (g/100 g Gd_2_O_3_)	I/I_0_ *	∑_t_ (cm^−1^)	∑_t/ρ_ (cm^2^/g)	HVL (cm)
0	0.72 ± 0.01	1.89 ± 0.08	1.70 ± 0.08	0.37 ± 0.02
5	0.47 ± 0.01	4.23 ± 0.03	3.80 ± 0.03	0.16 ± 0.01
10	0.65 ± 0.02	2.35 ± 0.20	2.08 ± 0.18	0.30 ± 0.03
15	0.65 ± 0.01	2.35 ± 0.07	2.08 ± 0.06	0.30 ± 0.01
20	0.56 ± 0.01	3.35 ± 0.14	3.02 ± 0.12	0.21 ± 0.01

* Values of I/I_0_ were determined using 2 mm thick samples.

**Table 5:** Mechanical properties, consisting of tensile modulus, tensile strength, elongation at break, and hardness (Shore D), of 20 wt% Gd_2_O_3_/r-HDPE composites, with varying silane (KBE903) contents (0, 5, 10, 15, and 20 g/100 g Gd_2_O_3_). The values are shown as the mean ± standard deviation of the mean.

Silane Content (g/100 g Gd_2_O_3_)	Tensile Modulus (MPa)	Tensile Strength (MPa)	Elongation at Break (%)	Hardness (Shore D)
0	154.6 ± 4.3	17.7 ± 0.3	35.0 ± 2.4	54 ± 3
5	173.4 ± 14.5	19.6 ± 0.4	51.6 ± 2.3	53 ± 4
10	65.6 ± 10.0	15.6 ± 2.4	20.9 ± 3.6	53 ± 3
15	100.5 ± 27.8	16.0 ± 1.5	26.3 ± 3.3	55 ± 2
20	69.7 ± 8.9	17.3 ± 1.5	24.7 ± 4.6	53 ± 3

**Table 6:** Densities and elemental compositions of Gd_2_O_3_/r-HDPE composites, with varying Gd_2_O_3_ contents (0, 5, 10, 15, and 20 wt%). The values are shown as the mean ± standard deviation of the mean.

Gd_2_O_3_ Content (wt%)	Density (g/cm^3^)		Elemental Composition (% by Weight)			
	Measured	Theoretical	C	O	Si	Gd
0	0.93 ± 0.01	0.93	96.35 ± 1.08	3.65 ± 1.08	n/a	n/a
5	0.97 ± 0.02	0.97	89.60 ± 1.51	3.44 ± 0.43	0.07 ± 0.02	6.89 ± 1.25
10	1.03 ± 0.01	1.02	85.30 ± 2.10	4.39 ± 1.17	0.10 ± 0.09	10.21 ± 1.16
15	1.09 ± 0.01	1.07	80.12 ± 3.31	3.96 ± 0.40	0.13 ± 0.10	15.79 ± 2.87
20	1.12 ± 0.01	1.13	74.51 ± 2.60	5.25 ± 0.27	0.26 ± 0.05	19.98 ± 2.79

**Table 7:** Remaining ash at 600 °C of Gd_2_O_3_/r-HDPE composites with varying Gd_2_O_3_ contents, determined through TGA.

Gd_2_O_3_ Content (wt%)	Remaining Ash at 600 °C (%)
0	2.0
5	5.7
10	10.4
15	14.2
20	16.5

**Table 8:** Degrees of crystallinity (%XC) for Gd_2_O_3_/r-HDPE composites, determined through XRD, with varying Gd_2_O_3_ contents (0, 5, 10, 15, and 20 wt%). The values are shown as the mean ± standard deviation of the mean.

Gd_2_O_3_ Content (wt%)	Degree of Crystallinity (%)
0	38.6 ± 4.5
5	53.6 ± 2.9
10	51.4 ± 4.4
15	50.2 ± 2.5
20	50.4 ± 2.2

**Table 9:** Comparative thermal-neutron-shielding and mechanical properties of 5 wt% Gd_2_O_3_/r-HDPE composites in this work and commercial PE products containing 5 wt% and 15 wt% of boron (B).

Property	5 wt% Gd_2_O_3_/r-HDPE	Borated PE **		Standard of Testing
		5 wt%	15 wt%	
Thermal-neutron-shielding properties *				
Total macroscopic cross section (cm^−1^)	4.43	0.36	0.60	–
Mass attenuation coefficient (cm^2^/g)	4.56	0.41	0.57	–
Half-value layer (cm)	0.16	1.94	1.15	–
Tenth value layer (cm)	0.52	6.43	3.80	–
Mechanical properties				
Tensile modulus (MPa)	185.5	>700	766.7	ASTM D638
Tensile strength (MPa)	20.9	>20	16.6	ASTM D638
Elongation at break (%)	157	–	4	ASTM D638
Hardness (Shore D)	60	>63	70	ASTM D2240

* Thermal neutron shielding measurements were carried out according to the procedure described in Section 2.4.1. ** The mechanical properties of borated PE products were obtained from provided specifications in their respective datasheets.

## Data Availability

Data will be made available on request (due to privacy).

## Supplementary Materials

The following are available online at https://www.mdpi.com/article/10.3390/polym16081139/s1: Figure S1: XRD spectra of Gd_2_O_3_/r-HDPE composites containing varying Gd_2_O_3_ contents of 0–20 wt%. The dotted red lines represent peak positions used for the calculation of crystallinity.
