# Supplementary material for: Highly Efficient and Eco-Friendly Thermal-Neutron-Shielding Materials Based on Recycled High-Density Polyethylene and Gadolinium Oxide Composites
Source: Polymers (Basel). 2024 Apr 18;16(8):1139. doi: 10.3390/polym16081139 (PMC11054564; doi:10.3390/polym16081139)
Supplement: Supplementary file 1 [file polymers-16-01139-s001.zip › polymers-2928224-supplementary.pdf]

Supplementary Materials

Highly efficient and eco-friendly thermal-neutron-shielding materials based on recycled high-density polyethylene and gadolinium oxide composites

Donruedee Toyen [1,2], Ekachai Wimolmala [3], Kasinee Hemvichian [4], Pattra Lertsarawut [4] and Kiadtisak Saenboonruang [2,5,6*]

[1] Department of Materials Science, Faculty of Science, Kasetsart University, Bangkok 10900, Thailand; donruedee.toyen@ku.th (D.T)

[2] Special Research Unit of Radiation Technology for Advanced Materials (RTAM), Faculty of Science, Kasetsart University, Bangkok 10900, Thailand

[3] Polymer PROcessing and Flow (P-PROF) Research Group, Division of Materials Technology, School of Energy, Environment and Materials, King Mongkut's University of Technology Thonburi, Bangkok 10140, Thailand; ekachai.wim@kmutt.ac.th (E.W.)

[4] Nuclear Technology Research and Development Center, Thailand Institute of Nuclear Technology (Public Organization), Nakhon Nayok 26120, Thailand; kasinee@tint.or.th (K.H.); pattra@tint.or.th (P.L.)

[5] Department of Applied Radiation and Isotopes, Faculty of Science, Kasetsart University, Bangkok 10900, Thailand

[6] Specialized Center of Rubber and Polymer Materials in Agriculture and Industry (RPM), Faculty of Science, Kasetsart University, Bangkok 10900, Thailand

* Correspondence: kiadtisak.s@ku.th; Tel.: +662-562-5555 (ext. 646219)

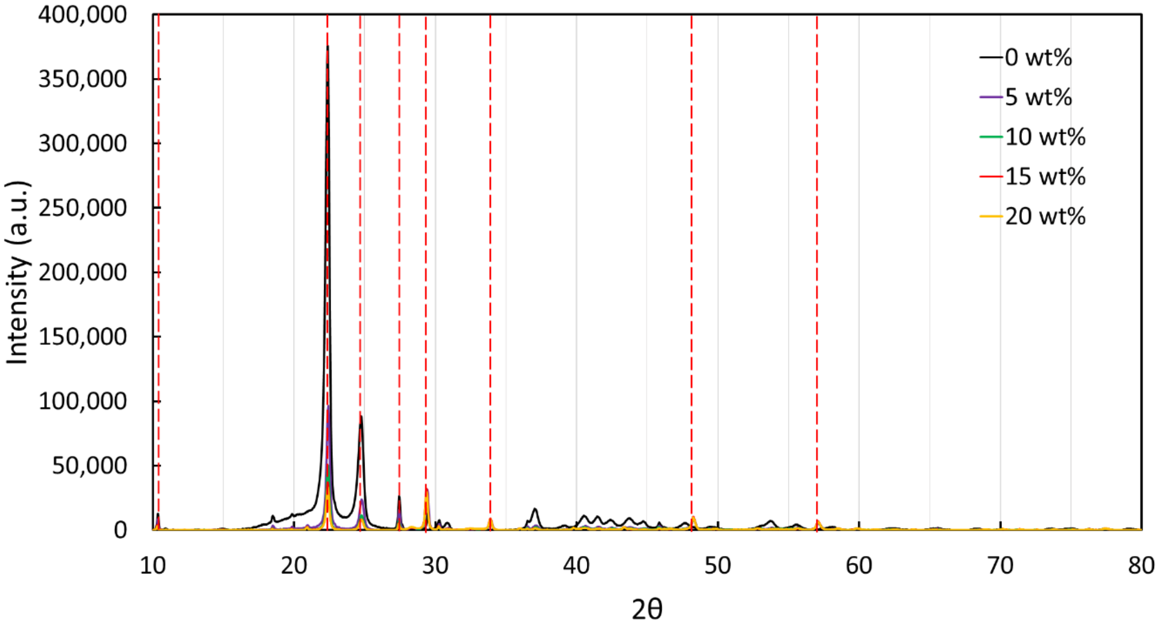

Figure S1. XRD spectra of Gd_2O_3/r-HDPE composites containing varying Gd_2O_3 contents of 0–20 wt%. The dotted red lines represent peak positions used for the calculation of crystallinity.
